# Implementation findings from a hybrid III implementation-effectiveness trial of the Diabetes Prevention Program (DPP) in the Veterans Health Administration (VHA)

**DOI:** 10.1186/s13012-017-0619-3

**Published:** 2017-07-26

**Authors:** Laura J. Damschroder, Caitlin M. Reardon, Mona AuYoung, Tannaz Moin, Santanu K. Datta, Jordan B. Sparks, Matthew L. Maciejewski, Nanette I. Steinle, Jane E. Weinreb, Maria Hughes, Lillian F. Pinault, Xinran M. Xiang, Charles Billington, Caroline R. Richardson

**Affiliations:** 1 Ann Arbor VA HSR&D/Center for Clinical Management Research, P.O. Box 130170, Ann Arbor, MI 48113-0170 USA; 2 VA Diabetes QUERI, Ann Arbor, MI USA; 3 VA Greater Los Angeles Healthcare System, 11301 Wilshire Blvd 3, Los Angeles, CA 90073 USA; 4 David Geffen School of Medicine, University of California, Los Angeles, CA USA; 5 Greater Los Angeles VA Health Services Research and Development (HSR&D) Center for Healthcare Innovation, Implementation and Policy, Los Angeles, CA USA; 6 Durham VA Medical Center HSR&D, 411 W Chapel Hill St, Suite 600, Durham, NC 27701 USA; 7 Duke University School of Medicine, Durham, NC USA; 8 VA Maryland Healthcare System, 10 North Greene St, Baltimore, MD 21201 USA; 9 University of Maryland School of Medicine, Baltimore, MD USA; 10 Department of Family Medicine, University of Michigan, Ann Arbor, MI USA; 11 Minneapolis VA Healthcare System, 1 Veterans Drive, Minneapolis, MN 55417 USA; 12 University of Minnesota Medical Center, Minneapolis, MN USA; 13 Scripps Translational Science Institute/The Scripps Research Institute, 10550 North Torrey Pines Road, Mail Drop: TRY-30, La Jolla, CA 92037 USA; 14 Louisiana State University Pediatric Neurology Program, 1542 Tulane Ave Rm 763, New Orleans, LA 70112 USA; 15 University of Michigan Department of Family Medicine, 1018 Fuller St, Ann Arbor, MI 48104 USA

**Keywords:** Implementation, Diabetes Prevention Program, Veterans, Consolidated Framework for Implementation Research, RE-AIM framework, Weight management, Pragmatic clinical trial

## Abstract

**Background:**

The Diabetes Prevention Program (DPP) is an effective lifestyle intervention to reduce incidence of type 2 diabetes. However, there are gaps in knowledge about how to implement DPP. The aim of this study was to evaluate *implementation* of DPP via assessment of a clinical demonstration in the Veterans Health Administration (VHA).

**Methods:**

A 12-month pragmatic clinical trial compared weight outcomes between the Veterans Affairs Diabetes Prevention Program (VA-DPP) and the usual care MOVE!® weight management program (MOVE!). Eligible participants had a body mass index (BMI) ≥30 kg/m^2^ (or BMI ≥ 25 kg/m^2^ with one obesity-related condition), prediabetes (glycosylated hemoglobin (HbA1c) 5.7–6.5% or fasting plasma glucose (FPG) 100–125 mg/dL), lived within 60 min of their VA site, and had not participated in a weight management program within the last year. Established evaluation and implementation frameworks were used to guide the implementation evaluation. Implementation barriers and facilitators, delivery fidelity, participant satisfaction, and implementation costs were assessed. Using micro-costing methods, costs for assessment of eligibility and scheduling and maintaining adherence per participant, as well as cost of delivery per session, were also assessed.

**Results:**

Several barriers and facilitators to Reach, Adoption, Implementation, Effectiveness and Maintenance were identified; barriers related to Reach were the largest challenge encountered by site teams. Fidelity was higher for VA-DPP delivery compared to MOVE! for five of seven domains assessed. Participant satisfaction was high in both programs, but higher in VA-DPP for most items. Based on micro-costing methods, cost of assessment for eligibility was $68/individual assessed, cost of scheduling and maintaining adherence was $328/participant, and cost of delivery was $101/session.

**Conclusions:**

Multi-faceted strategies are needed to reach targeted participants and successfully implement DPP. Costs for assessing patients for eligibility need to be carefully considered while still maximizing reach to the targeted population.

## Background

Incidence of type 2 diabetes (referred to as diabetes throughout) was reduced so dramatically (by 58%) in a landmark trial of the Diabetes Prevention Program (DPP) in the USA that the trial was stopped early in 2001 [1–4]. Since the original trial, many large-scale translations around the world, including Finland, Australia, China, and India, have successfully reduced onset of diabetes among patients with prediabetes and/or predictors like weight loss [5].

**Fig. 1:**
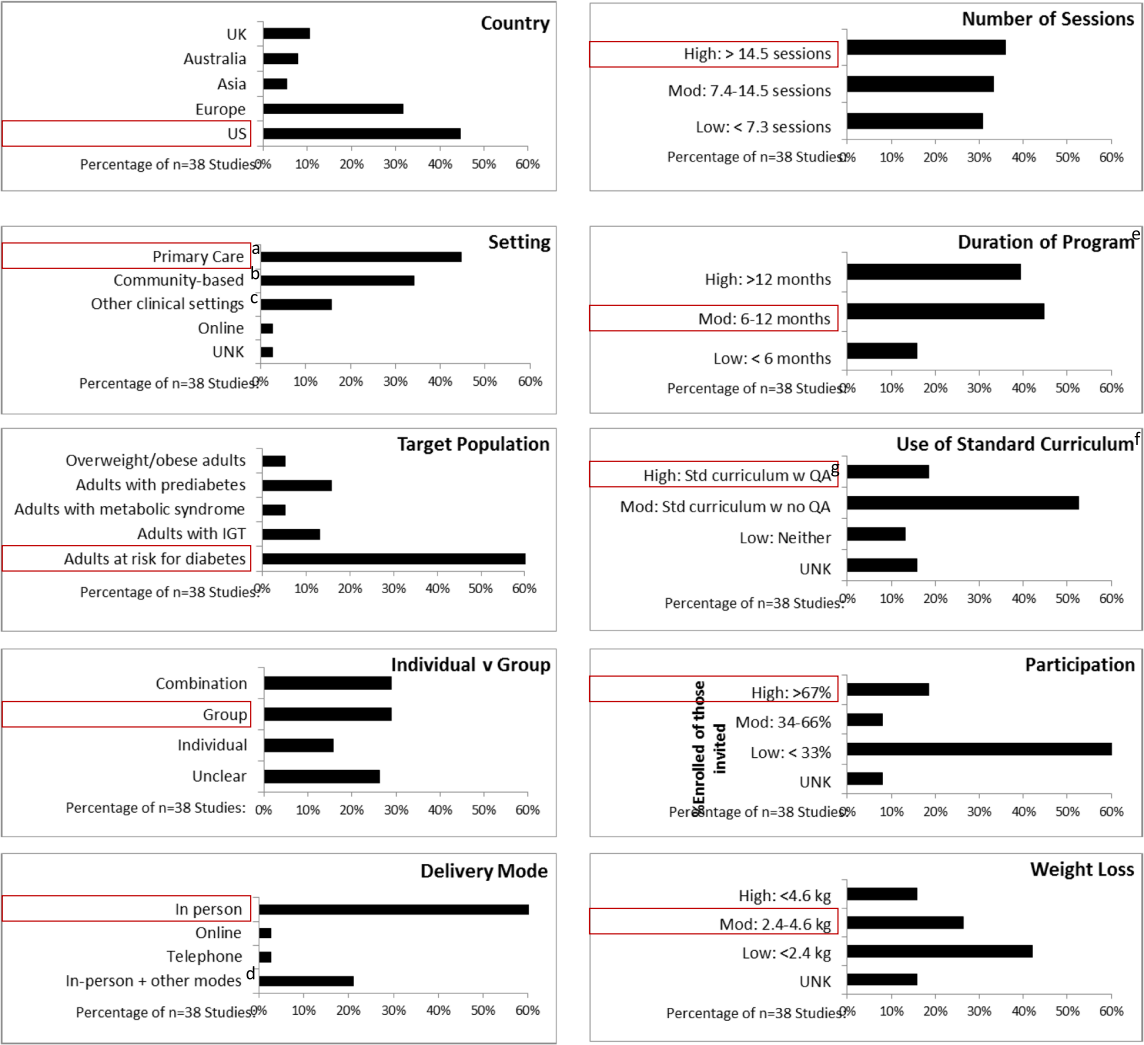
DPP characteristics reported by Aziz et al.’s systematic review. A red box indicates category for VA-DPP. ^a^Workplace and primary care settings. ^b^Community, church, YMCA, various venues, leisure, and community settings. ^c^Health care facilities, outpatient settings, hospitals. ^d^Other modes include telephone, fax, text, email, online. ^e^Thirty-nine studies reported because one study reported low and high sites. ^f^Standard curriculum = delivery of DPP following a standard curriculum. ^g^QA = quality assurance = use of measures to monitor implementation

To gain an overall perspective of the effectiveness of diabetes prevention studies, Balk et al. conducted a review of 53 studies (72% of which were randomized controlled trials), evaluating 66 combined diet and physical activity programs (41% of which were based on DPP), and concluded that such programs are effective in reducing incidence of diabetes, body weight, and FPG [6]. Programs varied widely in design: ranging from 3 to 72 months in duration and from no contacts (virtual contacts only) up to 72 contacts with participants. The review concluded that more intensive programs appeared to yield more positive outcomes, but there was little insight into how other program characteristics may contribute to outcomes. Since these studies focused on patient outcomes, they also provided no insight into how to successfully implement these interventions. Aziz et al. partially filled this gap with their systematic review of 38 studies with the goal of identifying factors leading to successful implementation of DPP in “real-world” settings [7] using the penetration, implementation, participation, and effectiveness (PIPE) impact metric. Figure 1 shows the distribution of program characteristics for eight dimensions and two outcomes: a wide range of program designs and durations have been implemented within clinical and community settings around the world. Across this diverse array of studies, two thirds reported low participation and 42% reported low weight loss (<4.6 kg) [7]. Both reviews by Aziz et al. and Balk et al. concluded that more intensive programs may enhance weight loss outcomes. However, Aziz et al. stress that even modest weight loss can have significant population-level impact if a high proportion of high-risk individuals participate in the program [7]. This broader view of impact is essential for policy- and other decision-making.

Kahn and Davidson [8] highlighted the dearth of real-world confirmation of the remarkable clinical outcomes reported by the original DPP study [4]. The Balk et al. review of DPP-like programs reported significant reduction in diabetes incidence (0.59; 95% CI, 0.52–0.66, based on 16 (30%) of 53 studies that reported this outcome) [6]. Aziz et al. reported “moderate” or “high” risk reduction for 7 (18%) of 38 studies; this outcome was “unknown” for the remaining 31 (82%) of studies [7]. The low proportion of studies reporting these clinical outcomes may indicate potential reporting bias toward positive results; on the other hand, these downstream effects are more challenging to assess within clinical settings outside of highly controlled lengthy clinical trials. Despite the “voltage drop” [9] often seen in outcomes within real-world settings, definitive guideline statements have been disseminated recommending DPP around the world [10–12]. Despite large-scale population-based availability of DPP in some countries (Finland being a notable leader [13]), within other countries, access to DPP is limited, in part because of the expense, lack of reimbursement by insurance or funding entities, and challenges of effectively implementing DPP across diverse uncontrolled settings and populations [14–17]. Within the USA, interest in implementing DPP is increasing because the US Centers of Medicare and Medicaid Services (CMS) plans to reimburse participation expenses for eligible individuals starting in 2018 [18]; over 1000 programs were listed in the Centers for Disease Control and Prevention (CDC) registry of certified programs [19] as of November 2016 compared to 500 in May 2014 [12].

In the USA, recognition status by the CDC Diabetes Prevention Recognition Status (DPRP) requires at least 50% of participants to have a diagnosis of prediabetes based on blood testing (or have documented history of gestational diabetes) [20], though risk assessments, administered by short surveys, are also available [21–30]. When eligible individuals are identified, outreach is needed to encourage participation in DPP and often clinical testing is used to verify diagnosis. Thus, significant challenges remain: designing and reliably executing robust approaches to identify, enroll, and engage individuals at high risk for diabetes.

Within VA, the national policy office responsible for prevention efforts, the National Center for Health Promotion and Disease Prevention (NCP), commissioned a pragmatic trial to demonstrate impact and feasibility of implementing DPP (VA-DPP) in VHA, in the context of the already existing MOVE!® weight management program (MOVE!) [31]. This clinical demonstration was conducted in three geographically diverse medical centers [31]. Candidate participants included patients with prediabetes (HbA1c 5.7–6.4% or FPG 100–125 mg/dL) who lived within 60 min of a demonstration site, were obese (BMI > 30 kg/m2) or overweight (BMI 25–30 kg/m2) with diagnosis of an obesity-related condition (e.g., hypertension, diabetes), and attended a MOVE! orientation session. Eligibility criteria were confirmed clinically; participants who did not have a HbA1c or FPG within prior 6 months were invited to have HbA1c screening, which is aligned with CDC DPP and national care guidelines [32, 33]. Patients for whom anti-glycemic medication (including metformin) was documented within their electronic health record in the last 6 months and those with contraindications to uptake of intensive lifestyle change were excluded.

VA-DPP characteristics are indicated in comparison to the Aziz et al. review in Fig. 1. In an intention-to-treat analysis, participants in two study arms (VA-DPP, usual care weight management program) experienced significant weight loss (*p* < 0.001). Participants in VA-DPP lost significantly more weight at 6 months compared to those in MOVE! (4.1 vs. 1.9 kg; *p* < 0.001) but the difference between programs was no longer statistically significant (3.4 vs. 2.0 kg, *p* = 0.16) at 12 months [34]. Reach was higher for VA-DPP compared to MOVE!: more participants assigned to VA-DPP completed at least one session (73%) compared to those assigned to MOVE! (58%; *p* = 0.002) [34]. VA-DPP also had higher rates of participation, e.g., 42.5% completed at least eight sessions compared to 31% for MOVE!. Neither VA-DPP nor MOVE! resulted in changed HbA1c compared to baseline [34]; this trial was not powered to examine differences in diabetes incidence.

This evaluation was guided by the Reach, Effectiveness, Adoption, Implementation, and Maintenance (RE-AIM) framework, which was designed to inform translation of evidence-based programs into practice and heighten public health impact by examining impact on domains beyond clinical outcomes [35, 36]. RE-AIM includes five domains: (1) Reach, the proportion of the targeted population willing to participate in the intervention; (2) Effectiveness, the impact of the intervention on key outcomes; (3) Adoption, the proportion of organizations and individuals within organizations who are willing to initiate the intervention within their setting; (4) Implementation, the fidelity and cost of delivery at the setting level; and (5) Maintenance, the extent to which the intervention is sustained over the longer term at the setting and individual levels. The aim of this study was to identify prevalent contextual factors that may have influenced outcomes within each RE-AIM domain.

## Methods

### Study design

The current study focuses specifically on implementation experiences within a pragmatic hybrid III effectiveness-implementation trial of VA-DPP compared to MOVE! within three medical centers [31]. This type of study has a primary focus on implementation but also evaluates clinical outcomes [37] that were reported elsewhere [34]. This paper reports quantitative and qualitative findings related to implementation. This evaluation was approved by five Institutional Review Boards (IRBs) for each of the five research institutions involved in the evaluation (three demonstration sites, one national coordinating center, and one research collaboration site).

### Interventions

Details about the programs evaluated are provided elsewhere [31, 34]. Briefly, VA-DPP and MOVE! were group-based lifestyle interventions. Each VA-DPP was a 12-month program with 22 planned sessions delivered by a single CDC-certified coach in closed cohorts (i.e., participants started and finished the program with the same group). MOVE! was an 8-10-week program followed by monthly maintenance sessions delivered in open cohorts (i.e., participants started the program at any time in two of three sites) by a multi-disciplinary team.

### Implementation approach

The VA-DPP implementation strategy was guided by the Simpson et al. program change model [31, 38]. First, each site’s clinical champion worked to elicit site leadership commitment, documented by a formal memorandum of understanding (MOU). Second, VA-DPP coaches and team members were trained by the Diabetes Prevention Support Center (DPSC) [39, 40]. Third, each site adapted a protocol to implement VA-DPP, with special focus on how it would interface with MOVE! [13, 31]. Similar to other published implementation studies [41], coordinating center staff ensured a uniform approach was followed across the sites, assisted with planning and problem-solving, and helped to obtain and manage MOUs and IRB approvals.

### Assessment of outcomes in RE-AIM domains

Multiple data sources (e.g., staff interviews, site visits, participant questionnaires) and data types (qualitative, quantitative) were used to assess barriers and facilitators that affected RE-AIM domains, as well as fidelity, participant satisfaction, and cost. Additional details are provided in the following sections.

#### Barriers and facilitators: Reach, Effectiveness, Adoption, Implementation, and Maintenance

Semi-structured interviews were conducted with VA-DPP team members during the early stages of implementation (*n* = 15) and after the enrollment ended (*n* = 23) either by phone or face to face during site visits. A purposive sample of other site staff and clinicians was also asked to participate in interviews. Interview guides were based on the Consolidated Framework for Implementation Research (CFIR) and are published elsewhere [31]. The CFIR was operationalized as a codebook for qualitative analysis to understand how contextual factors influence RE-AIM domains [31]. The CFIR describes 39 constructs across five domains that can be used to systematically assess and articulate contextual factors that may influence program implementation: (1) intervention characteristics (e.g., adaptability); (2) outer setting (e.g., external policies and incentives); (3) inner setting (e.g., leadership engagement); (4) individual characteristics (e.g., self-efficacy); and (5) process (e.g., planning) [42]. All interviews were audiorecorded and transcribed verbatim. In addition, site visit notes, meeting minutes, and emails between the coordinating center and sites were included in qualitative analyses to provide additional information about ongoing implementation processes.

Using published methods [43], two analysts independently coded and assigned ratings based on qualitative data. Differences in coding and rating were resolved through consensus. To enable comparison within and between sites, a comprehensive matrix of ratings by construct and site was developed [44]. This approach identified constructs that, from the perspective of the program teams, influenced implementation outcomes. NVivo® Version 10 software was utilized to aid coding and analysis [45].

#### Delivery fidelity

Corresponding sessions of VA-DPP and MOVE! (two sessions from each program with similar content) were assessed for delivery fidelity. At each site, the VA-DPP coordinator or other team member attended five to seven sessions and used a pre-specified checklist to rate various fidelity domains [46]. This analysis includes fidelity ratings of delivery of educational content, review of goal progress, goal setting, group cohesion, and coaching characteristics (managed the session, stayed on track, and created a supportive and empathetic environment (Table 1)). Previous work indicated that items related to goal progress review and coach delivery characteristics (Table 1) were associated with weight loss [46, 47]. Raters used a Likert scale (1 = strongly disagree to 7 = strongly agree) and provided optional open-ended comments. Factor analyses were conducted to confirm the appropriate groupings of items within domains, and Cronbach’s alpha was calculated for each domain to determine internal reliability. *T* tests were used to compare mean fidelity ratings between VA-DPP and MOVE!.

**Table 1:** VA-DPP coordinator ratings of fidelity for delivery of VA-DPP and MOVE!

	VA-DPP (*n* = 37)	MOVE! (*n* = 34)	*p* ^b^
Question^a^	Mean (SD)		
Delivery of educational content			
Coach elicited discussion of the educational content in order to help participants develop a self-management skill or change cognitions	6.62 (0.55)	6.00 (1.22)	0.0094
Goal setting, Cronbach’s alpha = 0.66^c^			
Type 2 diabetes prevention was discussed as a goal of the group	4.59 (2.20)	1.97 (1.70)	0.0000
Coach presented standardized goals (i.e., everyone had the same goal to complete the following week) to the participants and asked them to commit to a goal	5.59 (1.64)	3.62 (2.12)	0.0001
Review of goal progress, Cronbach’s alpha = 0.94			
Coach prompted review of goal progress and attainment^d^	6.41 (0.86)	4.76 (2.31)	0.0002
Coach elicited discussion of successes and challenges since the last session^d^	6.38 (0.82)	4.62 (2.32)	0.0001
Coach initiated problem-solving when necessary to address challenges since the last session^d^	6.44 (0.70)	5.21 (1.92)	0.0008
Group cohesion, Cronbach’s alpha = 0.81			
Group identity includes having a diagnosis of prediabetes	5.21 (1.63)	1.47 (0.90)	0.0000
Group members communicated easily with one another	6.10 (1.22)	5.35 (1.57)	0.0385
There were positive relationships among the group members	6.16 (1.10)	5.35 (1.54)	0.0184
Group members had a positive attitude toward the coach	6.74 (0.51)	6.06 (1.28)	0.0056
Group members participated actively in the group	6.41 (0.74)	5.59 (1.58)	0.0076
Coach characteristics, Cronbach’s alpha = 0.90			
Managed the session			
Coach came prepared and organized^d^	6.85 (0.36)	6.65 (0.65)	0.1091
Coach elicited clarification of participant engagement by seeking feedback about didactic content^d^	6.65 (0.60)	6.36 (0.96)	0.1510
Coach delivered didactic material in an engaging, matter of fact, and respectful way^d^	6.79 (0.41)	6.65 (0.69)	0.2900
Coach facilitated discussion and interaction using open-ended questions, affirmations, reflections, and summaries^d^	6.53 (0.61)	6.26 (0.96)	0.1814
Coach allocated time appropriately in order to cover the appropriate content focus points for the session^d^	6.62 (0.65)	6.47 (0.99)	0.4726
Coach supplied the necessary materials for the participants^d^	6.91 (0.29)	6.76 (0.43)	0.1026
Stayed on track			
Coach addressed process (tangential) issues but did not allow them to disrupt content agenda^d^	6.53 (0.71)	6.18 (1.06)	0.1105
Coach avoided delving too deeply into psychological issues^d^	6.74 (0.51)	6.50 (0.71)	0.1206
Created a supportive and empathetic environment			
Coach responded empathetically and accurately to participant behavior (verbal, nonverbal)^d^	6.85 (0.36)	6.59 (0.61)	0.0326

^a^1 = strongly disagree to 7 = strongly agree

^b^ *t* test

^c^Factor analyses were conducted to confirm the appropriate groupings of items within domains; Cronbach’s alpha was calculated for each domain to determine internal reliability

^d^Item was taken from published fidelity checklist [46]

#### Participant satisfaction

After 12 months, participant perspectives of their program were elicited through an administered survey that included questions about program satisfaction (Table 2). *T* tests compared mean ratings between VA-DPP and MOVE!. All analyses were conducted using Stata 13 [48].

**Table 2:** Participant program satisfaction at 12 months (*N* = 260)

Survey question	VA-DPP (*n* = 183) Mean (SD)	MOVE! (*n* = 77) Mean (SD)	*p* ^a^
Group preference			
If you had had the chance to switch into a different group working on diet and exercise, how would you have felt about switching? (1 = very much want to switch, 5 = very much want to stay)	3.75 (1.11)	3.19 (0.85)	0.0005
Group cohesion			
How well did you bond with your group members? (1 = did not bond, 2 = bonded a little, 3 = bonded very well)	2.45 (0.65)	2.20 (0.80)	0.0181
Participant satisfaction with coach			
When you had important questions to ask your coach, did you get answers you could understand? (1 = no, 2 = sometimes, 3 = always)	2.82 (0.44)	2.80 (0.41)	0.7002
Did you feel you were treated with respect and dignity during your group sessions? (1 = no, 2 = sometimes, 3 = always)	2.91 (0.33)	2.93 (0.31)	0.6653
Did you have confidence and trust in your coach? (1 = no, 2 = sometimes, 3 = always)	2.85 (0.43)	2.68 (0.60)	0.0233
Did your coach provide useful suggestions to help you overcome barriers in meeting your PA goals? (1 = no, 2 = sometimes, 3 = always)	2.81 (0.46)	2.57 (0.67)	0.0036
Did your coach provider meaningful feedback regarding your progress toward meeting your goals? (1 = no, 2 = sometimes, 3 = always)	2.80 (0.47)	2.48 (0.69)	0.0002
My coach motivated me to do my very best. (1 = strongly disagree, 5 = strongly agree)	4.48 (0.77)	4.0 (0.84)	0.0001

The overall response rate (67%) was similar to the response rate from VA-DPP (67%, *n* = 183) and MOVE! (68%, *n* = 77).

^a^Based on *t* tests for differences between MOVE! and VA- DPP fidelity ratings for sample of sessions delivered

#### Implementation costs

Details of the micro-costing approach used are described elsewhere [32]. Briefly, costs of implementing VA-DPP were estimated for two categories: (1) recruiting participants and implementing VA-DPP and (2) conducting VA-DPP sessions. VA-DPP coordinators and coaches at each site recorded time spent on a range of tasks: recruitment, administrative tasks, team meetings, session preparation, and session delivery. Total recruitment time was multiplied with per-minute wage rates to derive site-specific total cost. Total costs were divided by the number assessed and enrolled per site to derive per participant enrollment cost. VA-DPP sessions included costs of the labor inputs and participation rates associated with each group session. Appropriate per-minute wage rates were applied to the aggregated activity times. For delivering sessions, labor cost was divided by the number of participants who completed each session.

## Results

The following sections describe facets of the RE-AIM domains that were evaluated, including quantitative measures of delivery fidelity, participant satisfaction, and implementation costs. Common barriers and facilitators were evaluated for their influence on each of the RE-AIM domains. Each barrier and facilitator is demarcated with the associated CFIR construct (in parentheses) in order for readers to easily associate findings with the underlying theoretical framework. Because findings build on earlier published findings, there are additional citations to integrate the rich array of findings.

### Reach

#### Barriers and facilitators: recruitment for VA-DPP

As reported elsewhere, 1830 individuals attended a MOVE! orientation session, and 21% were eligible for the study [13, 34]. Overall, 11% were women and 48% were a racial/ethnic minority. The only demographic differences between VA-DPP and MOVE! participants were related to race/ethnicity; there were higher proportions of non-Hispanic black and non-Hispanic white VA-DPP participants, but a lower proportion of Hispanic participants (*p* = 0.04). Recruitment fell far short of the targeted sample size (*N* = 720 targeted; *N* = 387 enrolled; *N* = 386 in final analytic sample, due to a missing weight); there were 273 participants assigned to VA-DPP and 114 assigned to MOVE!. None of the sites achieved their recruitment goal due to several barriers.

A process to systematically identify individuals with prediabetes did not exist prior to VA-DPP implementation (Negative Compatibility). However, MOVE! had an existing obesity screening and referral process in place; VA-DPP recruitment relied on the MOVE! referral process. However, clinician referrals to MOVE! were lower than expected in all three sites. First, although health promotion and disease prevention was a high priority in VHA, clinicians felt they had limited time to discuss weight management and diabetes prevention with their patients. Patients frequently presented with multiple chronic and acute conditions which were a more immediate medical priority (Negative Relative Priority).

They don’t have time for [prediabetes], you know, they’re having a hard-enough time just dealing with the [patients with] out of control diabetes] […], it’s the old story about when you’re killing alligators, it’s hard to drain the swamp (Provider).

Second, some clinicians did not believe behavior modification programs were effective for their patients and were reluctant to refer patients (Negative Evidence Strength and Quality).

I’ve heard one physician who’s pretty vocal at the meetings say, “[MOVE!] doesn’t work, they re-gain the weight anyway.” A couple of physicians have said that actually. “Bariatric surgery’s the only thing that works.” So I don’t know that they even believe that a lifestyle program can work, and frankly some of our participants do well but a lot don’t (MOVE! Coordinator).

Third, primary care clinics at the three sites were overwhelmed by the major reorganization to align with VA’s version of PCMH (Negative Relative Priority). Fourth, one site had no formal MOVE! referral process and instead, patients were advised by their primary care and specialty providers to schedule a MOVE! orientation visit during the check-out process. This may have reduced one barrier (not requiring a provider referral) but introduced another when patients did not follow up (Negative Compatibility).

In addition, determining eligibility to participate in VA-DPP required a lab or point-of-care (POC) HbA1c or FPG test. One site had a POC testing procedure in place while another changed their electronic health record (EHR) to prompt clinicians to order an HbA1c test if they referred a participant to MOVE! (Positive Compatibility). The third site did not have either process in place; VA-DPP team members used a relatively labor-intensive process that involved evaluation based on EHR data or ordering a new HbA1c lab test for willing individuals (Negative Compatibility).

Lastly, the percentage of individuals referred to MOVE! with prediabetes was lower than expected; prevalence rates for normal glycemic status, prediabetes, and diabetes were 43% (*N* = 796), 22% (*N* = 404), and 28% (*N* = 504), respectively [34]. In effect, most patients referred to MOVE! were ineligible because they did not meet prediabetes criteria, which further contributed to unexpectedly low enrollment.

### Effectiveness

#### Barriers and facilitators: participation and attrition in VA-DPP

The percentage of participants who attended at least one session of VA-DPP was higher than for MOVE! (73.3 vs. 57.5%; *p* = 0.002). Although eligibility for VA-DPP was restricted to individuals who lived within 60 min of their sites, some participants still had transportation barriers (Negative Patient Needs and Resources):

I think it’s hard to come to a program every week in the middle of the day. […] [Site Three] is 5 miles away, that can be half an hour or 45 minutes and that’s not a doable thing for most people (Site Investigator).

Staff interviews revealed additional participation challenges such as time conflicts with work and family schedules or more seriously, insecure housing, unstable employment, and low income; these issues were more common for participants at two of the sites and affected participation in both MOVE! and VA-DPP.

VA-DPP participants also remained more engaged: more VA-DPP participants completed at least four sessions compared to MOVE! participants (57.5% VA-DPP; 42.5% MOVE!, *p* = 0.007) and more VA-DPP participants completed at least eight sessions compared to MOVE! participants (42.5% VA-DPP; 31% MOVE!, *p* = 0.035) [34]. VA-DPP team members described their VA-DPP participants having a positive experience (Positive Patient Needs and Resources):

They love coming to class, they like being around each other, they like hearing from each other, they like encouraging each other. […] I feel like overall the feedback has been pretty positive (VA-DPP Coach).

If asked, the MOVE! coordinators may have offered similar statements about their patients having positive experiences, but participant-reported satisfaction shows differences in satisfaction by program. Of the 387 participants across the two programs, 286 (74%) completed satisfaction questions 12 months after their baseline. Participants in VA-DPP reported higher levels of group preference (*p* = 0.0005) and group cohesion (*p* = 0.0181) than MOVE! participants (Table 2). They also reported modestly higher satisfaction with their coach than MOVE! participants for four of six items (Table 2).

### Adoption

#### Barriers and facilitators: facility adoption of VA-DPP

VA-DPP site leaders were familiar with the evidence base for VA-DPP (Positive Evidence Strength and Quality), which helped spark initial enthusiasm for having the program at their sites. In addition, these leaders acknowledged the benefits of targeting and engaging high-risk individuals in VA-DPP (Positive Relative Advantage), which they felt their existing MOVE! program did/could not do. However, one site leader expressed concerns about implementing the program, fearing bureaucratic challenges such as hiring new staff (Negative Structural Characteristics). Ultimately, all site directors signed an MOU as described above, visibly demonstrating their commitment to implementing VA-DPP (Positive Leadership Engagement). In addition, one site was motivated by the prospect of achieving CDC recognition for VA-DPP [32], while another was motivated by the enthusiastic endorsement of a national-level political leader (Positive External Policy and Incentives).

### Implementation

#### Fidelity and participant satisfaction

VA-DPP teams rated fidelity of program delivery for 71 sessions (37 sessions for VA-DPP, 34 sessions for MOVE!). Fidelity ratings were relatively high for both programs (Table 1) but were significantly higher for VA-DPP, except for comparable ratings for characteristics of the coaches leading the sessions.

#### Cost

Costs for labor to recruit participants and implement VA-DPP averaged $40,348 across the sites ($35,283–$54,877), which translated to $68 ($46–$97) per participant assessed and $330 ($210–$481) per participant identified to be eligible for VA-DPP.

Costs related to scheduling VA-DPP group sessions, sending out reminders to participants, and preparing for sessions averaged $28,462 across the sites ($16,889–$49,680), which translated to $328 ($156-$591) per participant.

Costs related to conducting VA-DPP sessions averaged $101 per group session conducted ($64–$177), which translated to a total cost of $2220 ($1410–$3889) for the planned 22 sessions for VA-DPP. Participants generated, on average, 57 completed sessions per site (means ranged from 19 to 119 across the sites); it cost $46 (range $41–$60) per participant per session.

#### Barriers and facilitators: implementation of VA-DPP

A strong VA-DPP team at each site supported by a central coordinating center was the single most important facilitator for successful implementation (Positive Engaging: VA-DPP Teams; Positive Engaging: External Change Agent). Team members strongly believed in VA-DPP to prevent diabetes, effectively solved problems, and felt a strong affinity with participants (Positive Engaging: VA-DPP Teams). In addition, they were familiar with previous DPP study findings (Positive Evidence Strength and Quality) and believed that VA-DPP had advantages over MOVE! (Positive Relative Advantage).

[VA-DPP] really focuses specifically on diabetes prevention, and not just weight loss in general, and I think those are […] two separate things, two separate goals (Site Investigator).

VA-DPP teams attended a 2-day training delivered by the DPSC (Positive Access to Knowledge and Information) that was well received.

I thought [the DPSC staff members] were excellent presenters, just very impressed…I was hoping to get them […] out here to do a workshop for us. That’s how effective I thought they were (VA-DPP Coordinator).

The DPSC also provided high-quality materials for VA-DPP teams and participants (Positive Design Quality and Packaging). Teams were further supported by the coordinating center (Positive Engaging: External Change Agent), which hosted bi-weekly meetings to provide new information and reflect on progress and problem-solve (Positive Access to Knowledge and Information; Positive Reflecting and Evaluating).

However, teams encountered several challenges. Some staff outside the VA-DPP team perceived VA-DPP as a competitor to MOVE! and questioned whether a new program was necessary (Negative Compatibility, Negative Relative Advantage).

If people are prediabetic, losing weight will help decrease their risk of becoming diabetic, so MOVE! in and of itself could serve as a Diabetes Prevention Program, although it’s not called that (MOVE! Coordinator).

In addition, sites were provided funding to hire staff, but hiring took months longer than expected and led to program delays (Negative Structural Characteristics). However, as one VA-DPP Coach noted, space was “…really far and away the biggest issue.” Sessions were rescheduled or canceled when the rooms were needed for other uses. Furthermore, VA-DPP session times were determined by available space, not by convenience for participants (Negative Available Resources). Although site directors signed an MOU, mid-level managers were largely disengaged and did not help teams resolve hiring or space issues (Negative Leadership Engagement). Lastly, VA-DPP coaches often struggled to cover all the content specified in the DPSC facilitation guides within hour-long sessions (Negative Design Quality and Packaging). However, the DPSC team clarified that coaches had latitude to adapt content to the needs of their groups (Positive Access to Knowledge and Information; Positive Reflecting and Evaluating).

### Maintenance

#### Barriers and facilitators: maintenance of VA-DPP

Two of the three sites continued to deliver VA-DPP as a separate program after the end of their VA-DPP demonstration period. At one site, monthly maintenance sessions were held after participants completed 12 months of VA-DPP followed by two separate projects focusing on peer-led and gender-specific VA-DPP implementation. One site did not sustain VA-DPP after funding ended due to insufficient resources to deliver VA-DPP and MOVE! concurrently (Negative Available Resources).

At the national level, however, NCP leaders recognized the early improvements in outcomes from VA-DPP compared to MOVE! [34] (Positive Evidence Strength and Quality). Subsequently, NCP updated guidance for MOVE! with VA-DPP features thought to contribute to greater weight loss (Positive Relative Advantage), including (1) having one consistent coach to lead all sessions; (2) offering closed cohorts; (3) providing 16 sessions within 6 months; and (4) aligning session topics more closely with VA-DPP topics (see www.move.va.gov/grpSessions.asp).

## Discussion

The challenge of implementing complex behavioral programs is well-recognized [6, 7, 49, 50]. The current findings point to the need for multi-level, multi-component strategies that include, for example, maximizing reach by carefully integrating processes to identify high-risk patients and engaging primary care providers. Conducting educational outreach and attempting to heighten clinical priority for preventing diabetes in the face of other competing demands is necessary to engage providers, the main source of referrals in the current study.

**Fig. 2:**
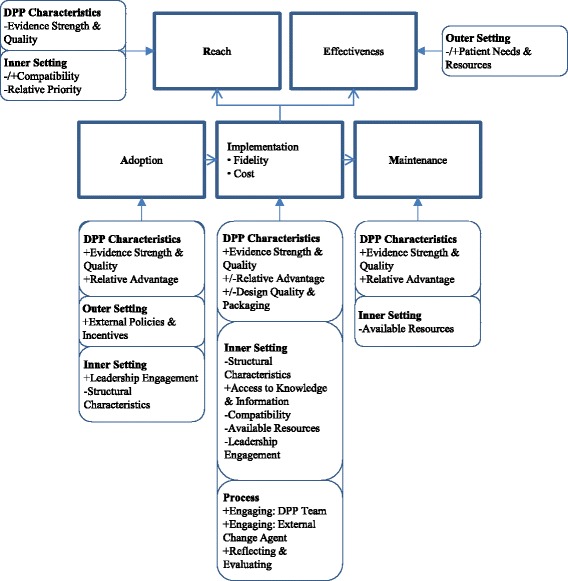
Conceptual framework: integration of CFIR contextual factors and RE-AIM domains

Many efforts to vastly scale up identification of high-risk individuals and encourage them to participate in DPP-like programs are underway in other countries [12, 51, 52]. Lindstrom and colleagues published the IMAGE toolkit, motivated by the premise that “Small changes in lifestyle will bring big changes in health…The time to act is now. ([53]; p. 537)” These motivating words are aimed at aligning partners toward a common high-priority goal around diabetes prevention. The toolkit describes foundational principles (e.g., engage partners from multiple strata including communities) and functions (e.g., reach out to high-risk individuals). The CDC also developed a toolkit for implementation [54]. Table 3 extends these recommendations by providing potential strategies aimed at optimizing outcomes within each of the five domains of RE-AIM based on barriers and facilitators shown in Fig. 2, which in turn reflect the experiences at the three study sites. These strategies are offered as hypotheses to be tested in a larger trial.

**Table 3:** Recommended strategies to address organizational level barriers as described by RE-AIM

Domain	Recommendations
Reach targeted participant population	• Design referral processes that are (1) compatible and integrated with existing clinical processes; (2) effective in identifying and engaging high-risk participants; and (3) easy to use • Engage clinicians who are the primary source of referrals through personal outreach and by providing easy-to-access, targeted information about DPP highlighting its (1) evidence base, (2) compatibility with local clinical processes, (3) advantages compared to status quo, and (4) organizational and clinical priority for diabetes prevention
Effectiveness of program	• Schedule sessions at a time and place convenient for participants
Adoption by clinical settings; evidenced by visible demonstration of commitment by executive leaders	• Target education and information to executive leadership about DPP including its (1) evidence base, (2) compatibility with clinical processes, (3) advantages compared to the status quo, and (4) organizational and clinical priority to inspire them to adopt the program • Obtain a formal agreement (e.g., memorandum of understanding), signed by executive leadership, to commit to implement DPP
Implementation with consistency (track costs and adaptations)	• Target education and information to mid-level and clinical managers about DPP including its (1) evidence base, (2) compatibility with clinical processes, (3) advantages compared to the status quo, and (4) organizational and clinical priority to inspire them to help implementation teams solve problems and review progress • Ensure adequate time to hire and train skilled and enthusiastic implementation leaders and coaches to deliver DPP • Provide high-quality materials to coaches and participants that can be used effectively to support delivery of effective coaching during sessions (e.g., see http://www.diabetesprevention.pitt.edu/). • Ensure adequate space availability for sessions • Schedule sessions at locations and times that are convenient to participants, e.g., in community settings outside of normal clinic hours
Maintenance of DPP in the clinical setting over time	• Effectively report on outcomes and other key benefits from the local DPP to executive leadership, managers, and clinicians (especially those who may refer their patients to DPP) to gain support for the program and build a robust referral network

Within VA, the usual care MOVE! weight management program can prevent incidence of diabetes [5, 55]. It is a relatively low-intensity program with no diabetes risk assessment. The present study evaluated a higher intensity intervention based on a CDC-recognized structured curriculum targeted to individuals with clinically verified prediabetes. This trial shows that having national and local leaders who are committed to diabetes prevention is a necessary, though far from sufficient, condition for successful implementation within a large healthcare system.

Reaching and engaging target populations is a universal challenge for comprehensive lifestyle programs across settings [7, 56]. There was relatively high participation in this trial (Fig. 1); more participants assigned to VA-DPP attended at least one session compared to participants assigned to MOVE!. In addition, unlike most behavioral intervention studies, which tend to over-represent non-Hispanic whites [57], 48% of VA-DPP participants identified as a racial/ethnic minority (compared to 25% of national VHA users) [58] and 11% of VA-DPP participants were women (compared to 8% of national VHA users [59]). Robust referral processes and networks to identify and engage participants at high risk are essential [13]. Once high-risk individuals have been referred, scheduling sessions at locations and times that are convenient to participants [60, 61] or offering online programming [62] may help bolster participation.

The single largest investment incurred for the VA-DPP demonstration was the total average $40,348 per site to assess eligibility and implement the program. In the current study, new clinical processes had to be designed and adapted to each local setting to accomplish the required clinical testing of HbA1c or FPG levels. Several countries have administered short self-assessment risk instruments [63, 64]. Individuals at high risk are then encouraged to enroll in a nearby community-based DPP-like program. The later the risk is identified, the more intensive the intervention should be [65]. The current study used more intensive risk identification based on clinical testing that relied on preexisting obesity treatment referral processes already in place. This approach may have resulted in later risk identification and contributed to the smaller than planned sample size but may justify the more intensive DPP. Number of referrals was lower during the demonstration period than in the years leading up to the demonstration, perhaps because of other organizational priorities during that time (e.g., implementation of PCMH during the demonstration) and there was an unexpectedly high prevalence of patients who already progressed to diabetes, especially compared to the general VHA patient population (43 vs. 25%, respectively) [34]. This illustrates the potential missed opportunities to identify and engage high-risk individuals *before* they progress to diabetes. Additionally, VA-DPP relied on primary care physicians to refer patients; however, there were cases where physicians did not believe in the effectiveness of lifestyle change programs for their patients.

Few studies report the extent to which DPP is delivered as designed, though stated use of a standard curriculum is a recognized contributor to greater weight loss [7, 66, 67]. The current study assessed fidelity of delivery. Though ratings of characteristics of the coaches delivering each of the programs (VA-DPP and MOVE!) were comparable (Table 1), VA-DPP had higher fidelity ratings for delivery of educational content, goal setting, review of goal progress, and group cohesion. Reinforcing this finding, VA-DPP participants reported higher satisfaction for six of eight program characteristics compared to MOVE!. Together, program differences in fidelity ratings [46] and participant satisfaction helped to identify factors that may explain higher participation rates for VA-DPP compared to MOVE!, as well as more weight loss for VA-DPP, [6, 7] at least in the short term. It is not clear what contributed to higher delivery fidelity for VA- DPP compared to MOVE!; specific characteristics of DPP as well as more recent training of DPP coaches may have contributed to this difference. Although there were no statistical differences in effectiveness of VA-DPP versus MOVE! at 12 months (3.4 vs. 2.0 kg lost, respectively; *p* = 0.16 [34]), the lack of statistical difference between groups may be in part due to the smaller than planned sample size.

The challenge of evaluating implementations of complex behavior change programs like DPP is well-recognized [68]. Use of a theoretical framework provides concepts and language that can be expressed consistently across diverse studies to aid comparisons and build knowledge about complex implementation processes [42, 69]. Based on current findings, Fig. 2 posits relationships between contextual factors and domains of RE-AIM based on common barriers and facilitators to VA-DPP implementation. These relationships are offered as hypotheses that require testing in other settings and within larger-scale implementations. Multi-faceted strategies that address multiple domains of context are needed to implement DPP in a way that maximizes outcomes within each RE-AIM domain (Table 3).

Though DPP has been shown to be cost-effective, including when delivered in a community setting [70, 71], health system and policy decision-makers will not implement programs like DPP without first knowing the upfront investment and ongoing delivery costs. Costs to deliver VA-DPP sessions averaged $101 per session. In the current study, attendance was highly variable across sites, across cohorts within each site, and across sessions within each cohort. While the average cost per participant per session was $46 ($1012 for 22 sessions) for VA-DPP, this cost could be as low as $12 per participant per session ($264 for 22 sessions), if the lowest-cost site ($1410) had the highest observed level of participation (119). This cost range was lower than cost reported for the original DPP (estimated costs of $1399 per person in the first year) [71] and are similar to those of a YMCA group-based DPP (estimated costs between $275 and $325 per person in the first year) [61]. In addition, administrative tasks related to preparing for each VA-DPP session, such as reminding participants to attend and planning the session, which averaged $328 per participant in the current study, must also be considered when allocating staff resources.

It is important to note limitations in this evaluation. First, only three academically affiliated VHA sites participated; therefore, implementation experiences reported may be unique to their respective settings. In addition, the study occurred during an organizational transformation to the VA’s model of PCMH within the primary care clinics, which may have contributed to lower than expected number of referrals. Second, fidelity ratings may be biased positively because they were determined by VA-DPP coordinators/coaches who rated their peers. However, this bias may be present for both programs because VA-DPP teams worked closely with MOVE! teams. It is important to note that comparison of ratings for VA-DPP and MOVE! was limited to a sample of program sessions and delivery components at these sites and not a comprehensive rating for all aspects of all sessions. Third, this effort was done as part of an effectiveness-implementation hybrid trial. National partners contributed funding for the clinical teams at each of the three facilities and did not assess diabetes incidence or clinical measures of cardiovascular disease or risks. When funding ended, two of the three sites were not able to maintain the program or continue screening for prediabetes. A strength of this study was that partners and local leaders deemed local activities, including eligibility screening, delivery of VA- DPP, and outcome assessments as part of a clinical quality improvement (QI) initiative that required no additional assessments for research purposes. Patient satisfaction was elicited via surveys funded by research. IRB approvals included use of data collected through local clinical QI activities. Fourth, an economic evaluation of usual care was not completed and intervention impacts on blood pressure and blood cholesterol were not assessed. Costs were estimated for eligibility screening, implementation, and delivery of only DPP.

## Conclusions

The comprehensive qualitative and quantitative findings from this pragmatic trial of VA-DPP reveal barriers and facilitators that influence overall program success. Findings suggest a number of strategies that may help support future real-world implementations of DPP, including gaining visible support from system and local leaders, highlighting the evidence base and benefits of DPP for key stakeholders including referring clinicians, and providing sufficient time and resources for high-quality staff training. As the need and demand for DPP increases, it is important to recognize, address, and leverage implementation process and contextual factors that contribute to maximum success of DPP.

## Abbreviations

BMI: Body mass index
CDC: Centers for Disease Control and Prevention
CFIR: Consolidated Framework for Implementation Research
CMS: Centers of Medicare and Medicaid Services
DPP: Diabetes Prevention Program
DPSC: Diabetes Prevention Support Center
EHR: Electronic health record
FPG: Fasting plasma glucose
HbA1c: Glycosylated hemoglobin
MOU: Memorandum of understanding
NCP: National Center for Health Promotion and Disease Prevention
PCMH: Patient-centered medical home
POC: Point-of-care
RE-AIM: Reach, Effectiveness, Adoption, Implementation and Maintenance framework
VA: Veterans Affairs
VA-DPP: Veterans Affairs Diabetes Prevention Program
VHA: Veterans Health Administration
